# Implementation of the Fast-track Protocol for Total Hip Arthroplasty in a Public Hospital in the State of São Paulo – Brazil

**DOI:** 10.1055/s-0043-1771489

**Published:** 2024-04-10

**Authors:** Leandro Gregorut Lima, Barbara Fialho Carvalho Sampaio, Marco Aurélio Silvério Neves, Alexandre Póvoa Barbosa, Victor Edmond Seid, Fernanda Degobbi T. Q. S. Lopes

**Affiliations:** 1 Arthron Serviços Médicos Especializados, São Paulo, SP, Brasil; 2 Departamento de Patologia, Faculdade de Medicina, Universidade de São Paulo, São Paulo, SP, Brasil; 3 Hospital Regional de São José dos Campos, Instituto Sócrates Guanaes, São José dos Campos, SP, Brasil; 4 Faculdade Albert Einstein, São Paulo, SP, Brasil

**Keywords:** arthroplasty, replacement, hip, arthroplasty, replacement, knee, clinical protocols

## Abstract

**Objective** Evaluate the results of the implementation of the Fast Track Protocol (FTP), a medical practice based on scientific evidence, for elective total hip arthroplasty surgery, mainly comparing the National Average Hospital Admission Rate of 7.1 days.

**Methods** 98 patients who underwent elective total hip arthroplasty surgery via the direct anterior approach, anterolateral approach and posterior approach were included in the FTP from December 2018 to March 2020, being followed up preoperatively, intraoperatively and immediately postoperatively.

**Results** The average length of hospital stay was 2.8 days, being 2.1 days for the direct anterior approach, 3.0 days for the anterolateral access approach and 4.1 days for the posterior access approach. The average surgery time was 90 minutes, 19 (19.39%) of the patients were referred to the ICU in the postoperative period, however, none of them underwent surgery using the direct anterior approach. We had no cases of deep vein thrombosis (DVT), pulmonary embolism (PTE) or neurological injury, 19 (19.39%) patients had postoperative bleeding requiring dressing change, 4 (4.08%) needed blood transfusion, 2 (2.04%) patients had implant instability, 1 (1.02%) patient had a fracture during surgery and 1 (1.02%) patient died of cardiac complications.

**Conclusion** FTP may be a viable alternative to reduce the length of stay and immediate postoperative complications for elective total hip arthroplasty surgery decreasing the length of stay of patients by 2 to 3 times when compared to the national average of 7.1 days.

## Introduction

In Brazil, there is a projection that in 2030, people over 60 years old who have a higher incidence of hip and knee osteoarthritis will be 19% of the Brazilian population or 42,122,847 people. 1

The growth of the elderly population also has consequences for the number of hip and knee arthroplasty surgeries performed in the SUS (Public Health System), in which there is an average annual increase of 3.3% and 8.7% respectively, 2 with 15,042 hospitalizations for hip arthroplasty in 2019, pre-pandemic due to COVID 19 according to the DataSus website.

Rapid Recovery Protocols originally come from Enhanced Recovery After Surgery (ERAS) or Fast Track Protocol (FTP) which was developed in the late 1990's by Dr. Henrik Kehlet as a strategy to reduce the length of hospital stay after major surgeries, but only from 2005 allowed a better recovery not only in quantitative terms, but also in qualitative terms. 3 4 According to the ERAS Society, the organization that initiated studies on FTP's for abdominal surgeries, there are about 20 precautions that influence the response to surgical stress and increase the patient's recovery speed after the procedure. Thus, an FTP team must include professionals who are engaged and experienced in surgery, anesthesia, nursing, physiotherapy, and nutrition. The team has primary responsibility for reviewing the available literature and formulating and delivering the appropriate protocol for their institution. 5 The implementation of the FTP in any hospital depends on the interaction and coordinated work of the multidisciplinary teams that are the pillars for the success of this journey of implementation and maintenance of the active protocol.

Taking into account that the Brazilian socioeconomic reality is that of a developing country and that the health system in Brazil is mostly represented by the SUS, which faces great financial difficulties and scarce resources, we adapted a FTP model to the reality of Brazilian public hospitals for elective total hip arthroplasty surgery performed in a public hospital in São Paulo - Brazil.

## Methodology

This work is a clinical research protocol that was conducted in compliance with all stipulations of this protocol, current national regulations, and guidelines established by the Document of the Americas and the ICH Guide to Good Clinical Practice. And it was approved by the Research Ethics Committee, under number CAAE: 30064919.6.0000.0068.

A prospective study carried out on 98 patients selected to electively undergo total hip arthroplasty surgery by anterolateral approach (AA), posterior approach (PA) and direct anterior approach (DAA), from December 2018 to March 2020.

Patients included with a diagnosis of hip osteoarthritis and/or necrosis of the femoral head (Ficat III or IV) and who agreed to sign the Informed Consent Form were eligible. Exclusion criteria: Crowe's dysplasia type 3 or 4; previous hip surgery; clinical inability or reluctance to participate in the study.

### Implementation Program

After performing the preoperative exams, the patients were referred for a preoperative consultation with the anesthesiologist for clinical evaluation and ASA classification. 6 If complementary exams or evaluations from other specialties were necessary, the patient was referred to the specialist, returning with the anesthesiologist for a final evaluation.

Once eligible for the surgery, the nursing team advised the hospitalization, explained details of the surgery and delivered an informative booklet with illustrations of the surgical procedure with the steps to be followed in the preoperative, intraoperative and postoperative periods. It was also explained to the patient that they would be discharged from the hospital as soon as their clinical condition was stable and safe according to established clinical parameters.

At that moment, the nutritionist guided the patient regarding pre-surgical caloric and nutritional intake, so that they could be hospitalized on the day of surgery in a favorable metabolic state, explaining the need to fast for 8 hours preoperatively and the supplemented abbreviated fasting methodology with maltodextrin 3 hours before surgery.

The patient was assessed by the physiotherapy team, with preoperative functional assessment scores. They guided the patient about strengthening exercises to be performed before surgery, so that the patient could maintain or even gain muscle mass in the hip and knee extensors, facilitating postoperative physical rehabilitation.

The criterion for choosing the surgical approach was based on the surgeon's experience. Each physician standardized the use of only one access route to the hip to be performed in all of their patients and the randomization occurred due to the random criterion for scheduling pre-surgical outpatient consultations with the surgeons.

Preferential anesthesia was spinal anesthesia without the use of morphine supplemented with pericapsular and periacetabular infiltration performed at the end of the procedure by the surgeon with 1ml per kilogram of the following solution: 70ml of Saline Sodium Chloride 0.9%, 30 ml of Ropivacaine 7.5mg/ml, 1 ml of Adrenaline 1mg/ml and 1 ml of Clonidine 150mcg/ml. If the patient had contraindication for spinal anesthesia, general anesthesia was performed.

The use of infusion of blood products was carefully evaluated, taking into account the surgeon's perception of blood loss during the surgical procedure, the calculation of the loss of aspirated fluids and the clinical conditions that were monitored by the anesthesiologist.

Postoperative referral to the ICU was restricted only to patients who presented hemodynamic instability, need for infusion of blood products or changes in cardiological monitoring during surgery.

The perception of pain during post-anesthetic recovery was assessed by the anesthetic team. Simple analgesics or anti-inflammatory drugs were prescribed and opioids were left only as a rescue option.

The patient was received from Post-Anesthetic Recovery, evaluated by the nursing team and the nutritionist prescribed the diet, avoiding prolonged fasting.

The physiotherapy team evaluated the patient and advised them on the need for early walking to avoid postoperative comorbidities such as DVT.

DVT prophylaxis was established with Enoxaparin Sodium 40mg subcutaneously once a day during the hospitalization period in the ward.

The surgeons held medical consultations together with the physiotherapist to provide support and initial gait guidelines on the same day or the day after the surgery. The criteria for starting gait were the patient's ability to sit up in bed, be able to transfer alone with the aid of the walker to the chair, stand up with the support of the walker without pain.

If a postoperative drain was used, it was preferably removed within 24 hours.

Hospital discharge guidelines were provided by the multidisciplinary team, emphasizing the following aspects: Encouraging the patient, family member and/or caregiver to talk about their concerns with home care, continuing rehabilitation at home with an emphasis on post-surgical restrictions such as not bending the hip more than 90 degrees, not crossing the operated leg over the other, not internally rotating the foot on the operated side.

Patients were released for hospital discharge if they met the following criteria: being pain-free, walking alone with or without the use of a walker, being able to sit up in bed alone, having spontaneously defecated and urinated, the dressing of the surgical incision had to be dry and clean, normal blood pressure, heart rate and temperature parameters.

Simple analgesics were prescribed for home, such as Dipyrone 1g every 6h if pain and Paracetamol 750mg every 6h if pain and if there was moderate or severe pain, Tramadol 50mg every 6h if pain was moderate or severe. DVT prophylaxis with Acetyl Salicylic Acid (ASA) 100mg orally for 30 days was also prescribed.

Evaluations regarding postoperative complications covered the immediate postoperative period up to 3 months of outpatient follow-up.

Weekly meetings between members of the multidisciplinary team were held with the intention of standardizing information and distributing tasks to be carried out in order to adapt FTP concepts to the Brazilian reality.

**Fig. 1:**
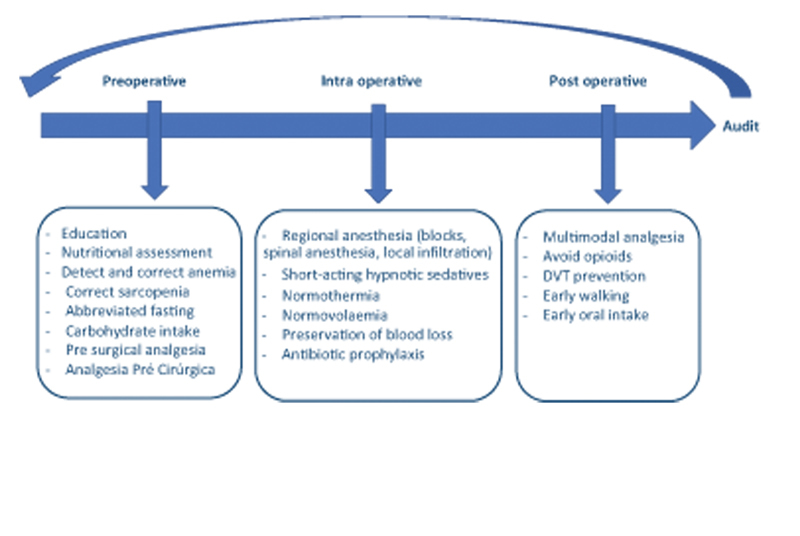
Pre, intra, and postoperative care for FTP implantation. (Adapted from Soffin EM, YaDeau JT. Enhanced recovery after surgery for primary hip and knee arthroplasty: a review of the evidence. Br J Anaesth. 2016 Dec;117).

During the meetings for the FTP to be implemented, questions were discussed together, establishing a goal of actions to be fulfilled. The personal engagement of each professional was essential for these actions to be implemented, since the communication of these new concepts to the respective professionals of each specialty involved was the responsibility of the professional participating in the FTP Implementation Group ( Fig. 1 ).

### Statistical analysis

Descriptive analyzes were carried out where the quantitative data that presented normal distribution were presented with means accompanied by the respective standard deviations. Data that did not show normal distribution were presented with medians and IQ interquartile ranges (25%-75%). Categorical variables were presented with frequency and percentage. The normal distribution in each group and the homogeneity of the variances between the groups were evaluated, respectively, with the Shapiro-Wilk test and the Levene test.

The Wilcoxon test for a sample and the Kruskal Wallis test were used for the analysis of numerical variables for multiple comparisons, the Dunn test was used.

To compare proportions, the chi-square or Fisher's exact test was used when necessary.

A statistical significance value less than or equal to 5% (p ≤ 0.05) was used for all analyzes.

Statistical modeling and tests were performed using SPSS software version 21.0.

## Results

**Fig. 2:**
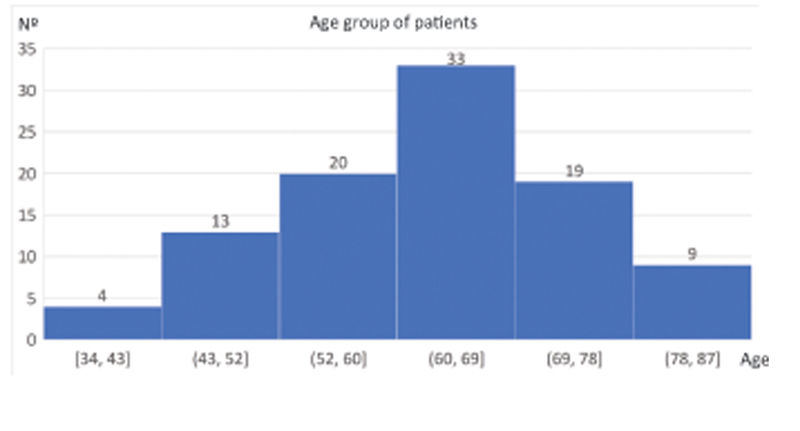
Age group of sample patients.

**Fig. 3:**
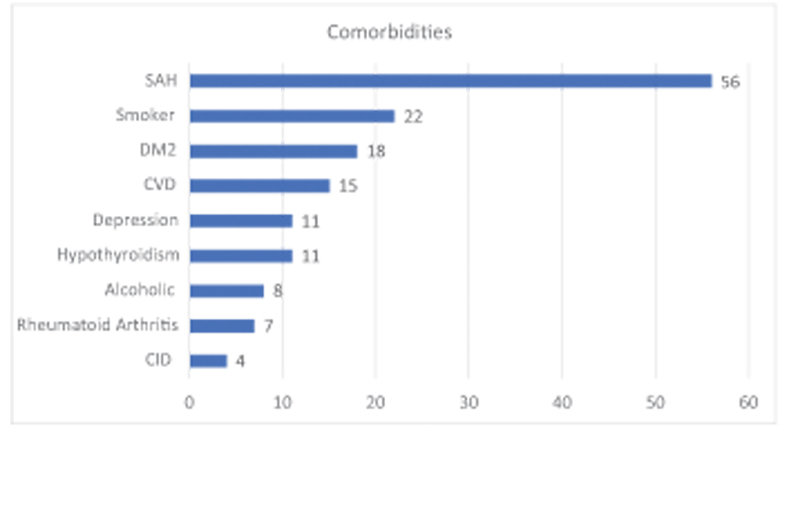
Incidence of comorbidities in the studied population. CID – Contagious Infectious Disease, CVD – Cardiovascular Disease, SAH – Systemic Arterial Hypertension, DM2–Type 2 Diabetes Mellitus.

All data from the study of 98 patients who underwent total hip arthroplasty from December 2018 to March 2020 were recorded in medical records and had a mean age of 62.8 years (34 to 81), 56 men and 43 women divided into the following age groups shown in Fig. 2 . 94 patients (95%) were operated on for primary osteoarthritis and 4 for osteoarthritis secondary to other pathologies, with the following associated comorbidities ( Fig. 3 ).

Of the 98 patients, 54 were operated on through AA access, 35 through the DAA and 9 through the PA.

**Fig. 4:**
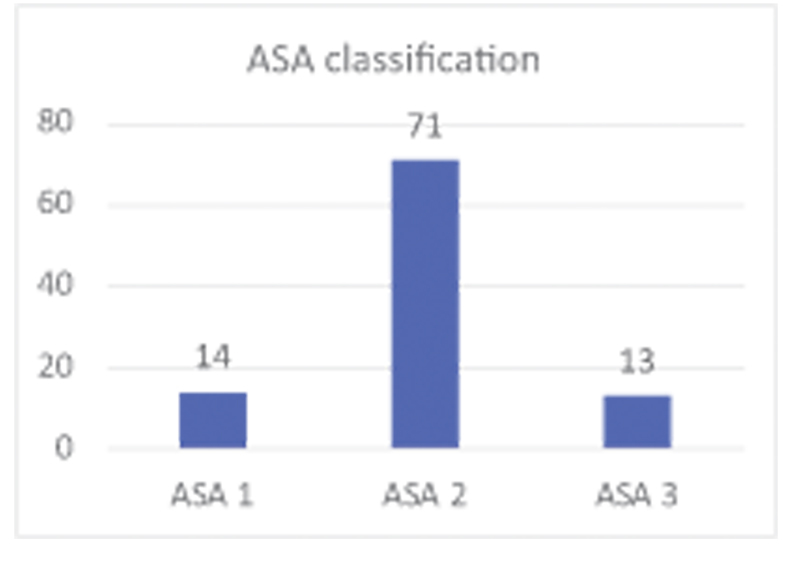
Distribution of patients according to the ASA classification of the American Society of Anesthesiology.

In the studied population, we had 14 (14.29%) of the patients classified as ASA1; 71 (72.45%) of the patients classified as ASA 2 and 13 (13.27%) of the patients classified as ASA 3 as shown in Fig. 4 .

**Fig. 5:**
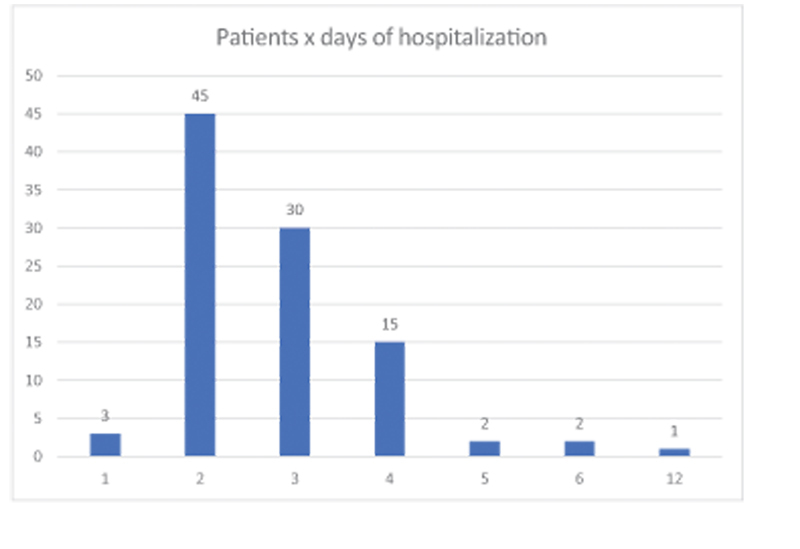
Hospital stay: 3 patients stayed 1 day, 45 patients stayed 2 days, 30 patients stayed 3 days, 15 patients stayed 4 days, 2 patients stayed 5 days, 2 patients stayed 6 days and 1 patient 12 days.

The average length of hospital stay was 2.8 days ( Fig. 4 ) with 3 (3.06%) patients staying 1 day, 45 (45.92%) staying 2 days, 30 (30.61%) staying 3 days, 15 (15.41%) staying 4 days, 2 (2.04%) 5 days, 2 (2.04%) 6 days and 1 (1.02%) staying 12 days according to Fig. 5 .

**Fig. 6:**
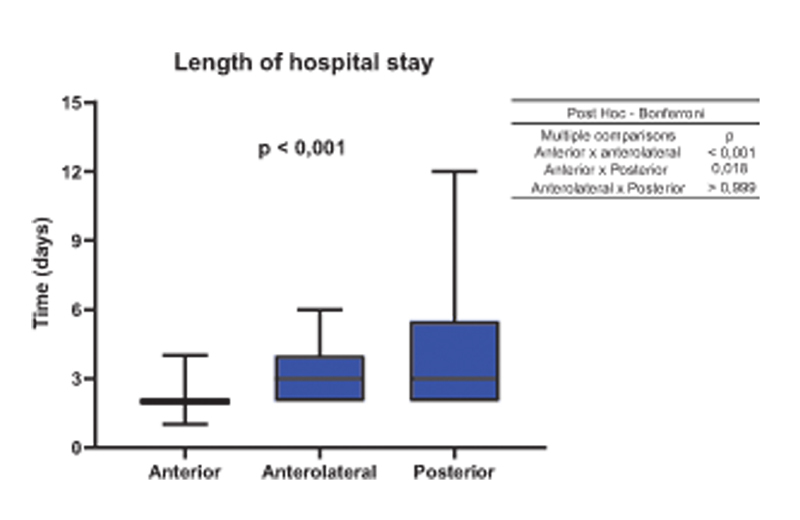
Length of hospital stay of 2.1 days for the anterior approach, 3.0 days for the anterolateral approach and 4.11 days for the posterior approach.

The average length of hospital stay for DAA was 2.1 days, 3 days for AA and 4.11 days for PA as seen in Fig. 6 .

The average surgery time was 90 minutes, 4 (4%) patients required transfusion of blood products, 19 (19%) of the patients were referred to the ICU in the postoperative period, with 14 (76%) remaining 1 day and 5 ( 26%) 2 days.

**Fig. 7:**
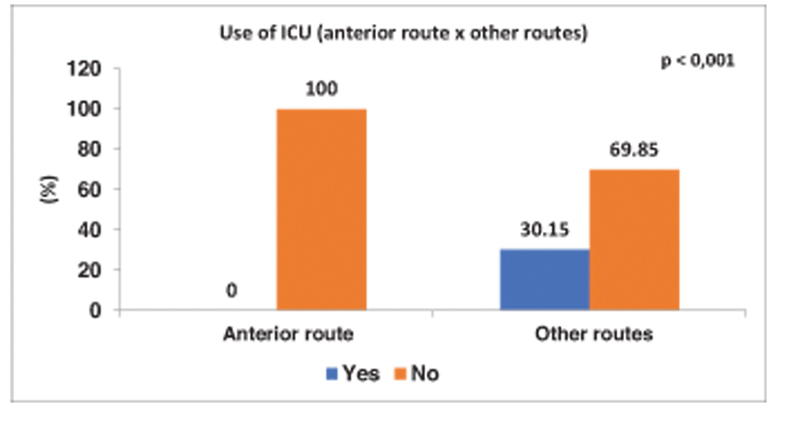
No patient operated via the anterior approach went to the ICU postoperatively. 30.15% of patients operated on by other approaches went to the ICU and 69.85% were not.

Of the 35 patients operated by DAA, none went to the ICU in the postoperative period, while 19 (30.15%) operated by other means went to the ICU, as shown in Fig. 7 .

**Fig. 8:**
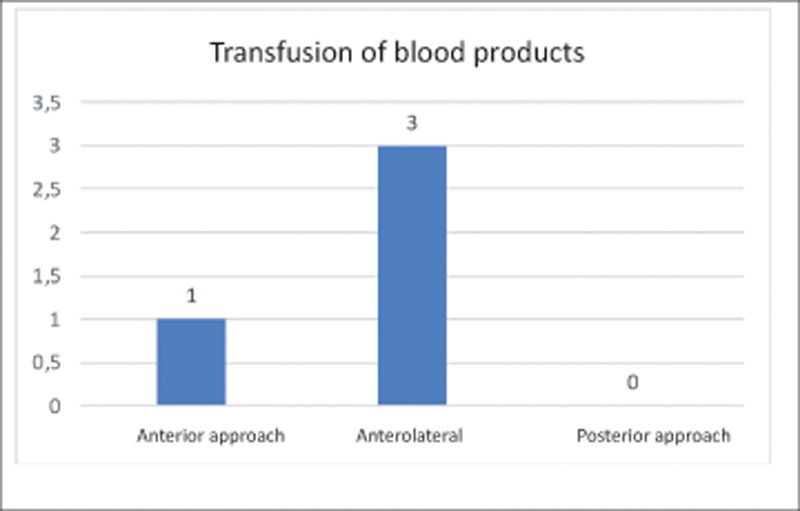
Of the total sample of 98 patients, 4 (4.08%) received transfusion of blood products, 1 (1.02%) operated through the anterior approach and 3 (3.06%) through the lateral approach.

Of the total sample of 98 patients, 4 (4.08%) required transfusion of blood products during the surgical procedure, 1 (1.02%) operated through DAA and 3 (3.06%) through AA. There were no patients who received transfusions after the end of the surgical procedure, as shown in Fig. 8 below.

During the hospitalization period, the stimulus for early walking was followed according to the methodology and all patients were discharged walking with the help of a walker.

**Fig. 9:**
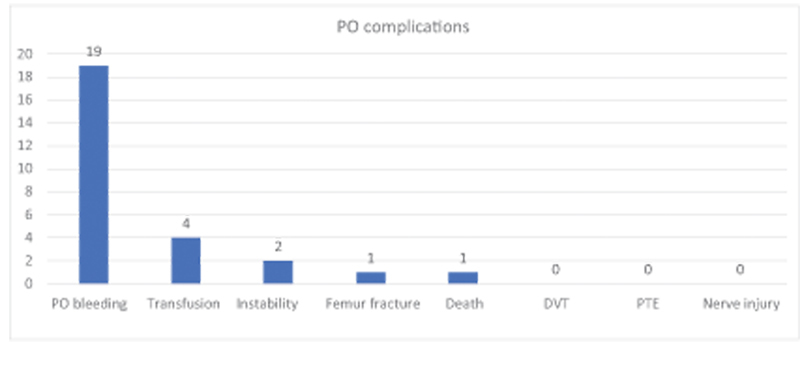
Total sample 98 patients, 19 (19.39%) of the patients had bleeding in the immediate postoperative period, 4 (4.08%) required transfusion of blood products, 2 (2.04%) had implant instability, 1 ( 1.02%) had a femur fracture, 1 (1.02%) died. There were no cases of Deep Vein Thrombosis (DVT), Pulmonary Thromboembolism (PTE) or Peripheral Nerve Injury.

As for immediate postoperative complications during the hospitalization period, we had no cases of deep vein thrombosis (DVT), pulmonary embolism (PTE) or neurological injury, 19 (19.39%) patients had postoperative bleeding requiring replacement of dressing, 4 (4.08%) needed blood transfusion, 2 (2.04%) patients had implant instability verified during hospitalization, 1 by anterolateral approach and 1 by a posterior approach, and were treated conservatively, 1 (1.02%) patient had a fracture during surgery, with femur cerclage being performed, and 1 (1.02%) patient died of cardiac complications, as shown in Fig. 9 .

**Fig. 10:**
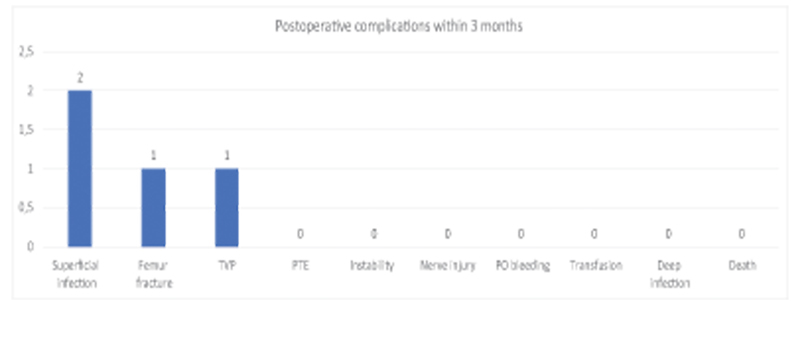
Postoperative complications within 3 months. We had 2 (2.04%) cases of superficial infection of the surgical wound, 1 (1.02%) of femur fractures and 1 (1.02%) case of DVT.

In the postoperative follow-up of up to 3 months, we had the following complications: 2 (2.04%) patients had a superficial infection in the access route treated with oral antibiotic therapy, 1 (1.02%) patient had a femur fracture without deviation due to a fall from own height 2 months after the initial surgical procedure and was treated conservatively and 1 (1.02%) patient had DVT diagnosed in the operated limb 15 days after the surgical procedure, however it was found that the patient did not take the prophylaxis recommended as can be seen in Fig. 10 .

## Discussion

Our overall length of hospital stay (LHS) was 2.8 days, 2.1 days for the anterior route, 3.0 days for the anterolateral route, and 4.1 days for the posterior route.

**Fig. 11:**
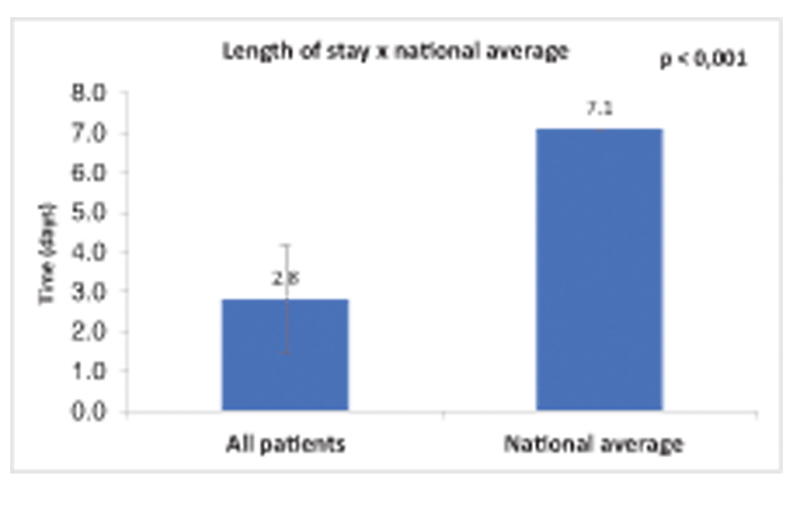
The length of stay for all patients in the sample was 2.8 days and the national average is 7.1 days (p < 0.001).

According to Marcio de Castro Ferreira, in his article “Total knee and hip arthroplasty: The worrying reality of care in the Brazilian Unified Health System”, the national average length of hospital stay for elective total hip arthroplasty is 7.1 days 2 ( Fig. 11 ).

Overall, there is little evidence to support the use of structured preoperative education to reduce postoperative adverse events, improve pain, facilitate functional recovery, or reduce length of stay. However, a significant reduction in preoperative anxiety can be achieved. Preoperative education may benefit more patients with depression, anxiety, unrealistic expectations or limited social support. 7 8 9

Decreased length of stay is consistently associated with the use of neuraxial anesthesia (anesthetic block) compared with the use of general anesthesia. In a multicenter retrospective study associating the use of general anesthesia with an 8.5-fold increased risk of moderate to severe postoperative pain and a 2.5-fold increased risk of persistent postoperative pain, for hip arthroplasty and knee. 10 11 These data provide additional motivation for the use of neuraxial anesthesia or plexus block.

Non-opioid spinal anesthesia supplemented with local infiltration analgesia (LIA) is a recent technique for early postoperative analgesia after knee and hip arthroplasty, and was administered by surgeons shortly after completion of the surgical procedure, providing 6–12 hours of pain relief so patients benefit from multimodal analgesia and oral opioids only if needed. 12 13

Our results with a total sample of 98 patients showed that 19 (19.39%) had bleeding in the immediate postoperative period with the need to change the surgical dressing, 4 (4.08%) required transfusion of blood products, 2 (2 .04%) had implant instability with signs of subluxation of the prosthesis, one patient operated via an anterolateral approach and one patient operated via a posterior approach, both of which were treated conservatively and followed up on an outpatient basis, 1 (1.02%) had a fracture of the femur during the surgical procedure, fixation with cerclage using steel wires was performed, 1 (1.02%) died of cardiac complications.

The results obtained corroborate Starks et al. who applied the FTP in patients undergoing total hip or knee arthroplasty, observed that the mortality rate for total knee arthroplasty decreased from 0.44 to 0.07%. Starks' work can be seen as an early model for FTP in orthopedic surgery. 5

Nutritional guidelines now allow fluid intake up to 3 hours before induction of anesthesia and 6-hour fasting for solid food. In addition to the reduced fasting time, the FTP recommends that patients consume up to 300 ml of a clear carbohydrate-rich drink 3 hours before surgery, with the aim of presenting the patient for surgery in a metabolically “fed” state, avoiding catabolism. 14 15 16 17

In our sample, we did not have any DVT or PTE events during the hospitalization period. Early mobilization is a key component of the FTP. Adverse physiological effects of prolonged bed rest include increased insulin resistance, myopathy, reduced lung function, impaired tissue oxygenation, and increased risk of pulmonary thromboembolism. 18 Pua et al. 19 demonstrated a significant reduction in length of stay of 1.8 days when patients walked within 24 hours of surgery. Early mobilization after knee arthroplasty is also associated with improved functional recovery and lower incidence of DVT. 20 21

The stimulus for early walking was followed according to the methodology criteria and all patients were discharged walking with the help of a walker and safety recommendations made by the physiotherapy and nursing team.

In our sample, only 4 (4.08%) patients received transfusion of blood products during the surgical procedure. A strategy that preserves the need for blood administration is crucial to the success of the FTP. Allogeneic blood transfusion is associated with immunomodulation and systemic volume overload. 22

Although initially adopted in orthopedic surgeries for primary hip and knee arthroplasties, the FTP has been increasingly applied to other orthopedic procedures, bringing benefits to patients and reducing procedure costs. 23 24 25 26 27

Recently, an article by the Mayo Clinic, an important reference hospital in orthopedic surgeries in the United States, reported the benefits obtained in arthroplasty surgeries reconciled with FTP, where patients undergoing surgery had fewer side effects related to opioids, fewer postoperative complications, shorter hospital stay and greater cost savings for the clinic. 28 29

We believe that the reasons for the early discharge of these clinically stable patients include several aspects, including the preoperative guidance that reduces anxiety about the procedure and establishes that it is not necessary to stay in hospital for a long period to recover properly, the use of the mixed anesthesia technique - spinal anesthesia and anesthesia by local infiltration, providing more comfort to the patient for a longer time, contributing for them to leave the acute inflammatory phase of the postoperative period - the adequate use of blood products, contributing to avoid a physiological lowering of the patient due to transfusion of exogenous elements, encouraging early mobilization contributing to the return of gastrointestinal functions, peripheral circulation of the limbs and deep circulation of the operated region, increasing tissue perfusion with oxygen and contributing to the reduction of the inflammatory process and finally the absence of complications such as PTE and DVT in the immediate postoperative period.

## Conclusion

The implementation of the FTP requires that a multidisciplinary team composed of surgeons, anesthesiologists, nurses, physiotherapists, nutritionists and social workers work together so that the steps are fulfilled and the needs are implemented, verified and constantly evaluated to reach an acceptable index, fulfilling all the planned actions.

The FTP only works well when all parties contribute to the overall result, and weekly follow-up and planning meetings, not only in the implementation phase, but also in daily execution, are essential for the project to be executed correctly and effectively.

The FTP has the ability to reduce the length of hospital stay, reduce the need for ICU use, use of blood products and high-cost analgesics, contributing to an early and more efficient recovery of the patient, as well as reducing hospital and social costs of returning this patient to their activities of daily living.

I draw attention to the effectiveness of the FTP in a Public Hospital and emphasize the importance of multidisciplinary work and data auditing to better substantiate the conclusions.
