# Supplementary material for: Implementation of the Fast-track Protocol for Total Hip Arthroplasty in a Public Hospital in the State of São Paulo – Brazil
Source: Rev Bras Ortop (Sao Paulo). 2024 Apr 10;59(2):e297–306. doi: 10.1055/s-0043-1771489 (PMC11006524; doi:10.1055/s-0043-1771489)
Supplement: Related texts

# Research article: Implantação do protocolo de recuperação rápida para artroplastia total do quadril em hospital público do estado de São Paulo – Brasil

**Keywords:** artroplastia do quadril, artroplastia do joelho, protocolos clínicos

## Abstract

**Objetivo** Avaliar os resultados da implantação do Protocolo de Recuperação Rápida (PRR), prática médica baseada em evidências científicas, para cirurgia eletiva de artroplastia total do quadril principalmente comparando à Taxa Média de Internação Hospitalar nacional de 7.1 dias.

**Métodos** 98 pacientes submetidos a cirurgia eletiva de artroplastia total do quadril pela via direta anterior, via anterolateral e via posterior foram incluídos no PRR no período de dezembro de 2018 a março de 2020 sendo acompanhados no pré-operatório, intraoperatório e pós-operatório imediato.

**Resultados** a Taxa Média de Permanência Hospitalar foi de 2,8 dias, sendo 2,1 dias para a Via de Acesso Anterior, 3,0 dias para via de acesso anterolateral e 4,1 dias para via de acesso posterior. O tempo médio de cirurgia foi de 90 minutos, 19 (19,39%) dos pacientes foram encaminhados à UTI no pós-operatório, no entanto nenhum deles operado pela via direta anterior. Não tivemos casos de trombose venosa profunda (TVP), embolia pulmonar (TEP) ou lesão neurológica, 19 (19,39%) pacientes tiveram sangramento pós-operatório com necessidade de troca de curativo, 4 (4,08%) necessidade de transfusão sanguínea, 2 (2,04%) pacientes apresentaram instabilidade do implante, 1(1,02%) paciente teve fratura durante a cirurgia e 1(1,02%) paciente faleceu por complicações cardíacas.

**Conclusão** O PRR pode ser uma alternativa viável para diminuir o tempo de internação e as complicações pós-operatórias imediatas para a cirurgia eletiva de artroplastia total do quadril diminuindo 2 a 3 vezes o tempo de internação dos pacientes quando comparado com a média nacional de 7,1 dias.

## Introdução

No Brasil existe uma projeção que em 2030, pessoas com mais de 60 anos que apresentam uma maior incidência de osteoartrose de quadril e joelho, serão 19% da população brasileira ou 42.122.847 pessoas. 1

O crescimento da população idosa também traz consequências para o número de cirurgias de artroplastia de quadril e joelho realizadas no SUS (Sistema Público de Saúde), em que há um aumento médio anual de 3,3% e 8,7% respectivamente, 2 com 15.042 internações por artroplastia de quadril em 2019, pré-pandemia por COVID 19 segundo o site do DataSus.

Os Protocolos de Recuperação Rápida provêm originalmente do Enhanced Recovery After Surgery (ERAS) ou Fast Track Protocol (FTP) que foi desenvolvido no final da década de 1990, pelo Dr. Henrik Kehlet como uma estratégia para diminuir o tempo de permanência hospitalar após cirurgias de grande porte, mas somente a partir de 2005 permitiu uma melhor recuperação não só em termos quantitativos, mas também em termos qualitativos. 3 4 Segundo a Sociedade ERAS, organização que iniciou os estudos sobre os PRR's para cirurgias abdominais, existem cerca de 20 cuidados que influenciam a resposta ao estresse cirúrgico e aumentam a velocidade de recuperação do paciente após o procedimento. Assim, uma equipe de PRR deve incluir profissionais engajados e experientes em cirurgia, anestesia, enfermagem, fisioterapia e nutrição. A equipe tem a responsabilidade primária de revisar a literatura disponível, formular e entregar o protocolo apropriado para sua instituição. 5 A concretização da implantação do PRR em qualquer instituição hospitalar depende da interação e do trabalho articulado das equipes multidisciplinares que são os pilares para o sucesso dessa jornada de implantação e manutenção do protocolo ativo.

Levando em conta que a realidade socioeconômica brasileira é a de um país em desenvolvimento e que o sistema de saúde no Brasil é majoritariamente representado pelo SUS, que enfrenta grandes dificuldades financeiras e escassez de recursos, adaptamos à realidade dos hospitais públicos brasileiros um modelo de PRR para cirurgia eletiva de artroplastia total de quadril realizada em um hospital público de São Paulo - Brasil.

## Metodologia

Este trabalho é um protocolo de pesquisa clínica que foi conduzido em conformidade com todas as estipulações deste protocolo, os regulamentos nacionais atuais e as diretrizes estabelecidas pelo Document of the Americas e o Guia de Boas Práticas Clínicas do ICH e foi aprovado pelo Comitê de Ética em Pesquisa, sob o número CAAE: 30064919.6.0000.0068.

Estudo prospectivo realizado em 98 pacientes selecionados para realizarem eletivamente a cirurgia de artroplastia total do quadril por abordagem anterolateral (AL), abordagem posterior (AP) e abordagem direta anterior (ADA), no período de dezembro de 2018 a março de 2020.

Foram elegíveis os pacientes incluídos com diagnóstico de osteoartrose do quadril e/ou necrose da cabeça femoral (Ficat III ou IV) e que concordaram com a assinatura do Termo de Consentimento Livre e Esclarecido. Critérios de exclusão: displasia de Crowe tipo 3 ou 4; cirurgia prévia de quadril; incapacidade clínica ou relutância em participar do estudo.

## Programa de Implantação

Após a realização dos exames pré-operatórios, os pacientes foram encaminhados para consulta pré-operatória com o anestesiologista para avaliação clínica e classificação ASA. 6 Caso fossem necessários exames complementares ou avaliações de outras especialidades, o paciente era encaminhado ao especialista, retornando com o anestesista para avaliação final.

Uma vez elegível para a cirurgia, o paciente era orientado pela equipe de enfermagem quanto à internação, explicava detalhes da cirurgia e entregava uma cartilha informativa com ilustrações do procedimento cirúrgico com as etapas que serão seguidas no pré-operatório, intra e pós-operatório. Também era explicado ao paciente que ele receberia alta hospitalar assim que sua condição clínica estivesse estável e segura de acordo com parâmetros clínicos estabelecidos.

Nesse momento, o nutricionista orientava o paciente quanto à ingestão calórica e nutricional pré-cirúrgica, para que fosse internado no dia da cirurgia em estado metabólico favorável, explicando a necessidade de realizar jejum pré-operatório de 8 horas e a metodologia de jejum abreviado suplementado com maltodrextrina 3 horas antes da cirurgia.

O paciente foi avaliado pela equipe de fisioterapia, com escores pré-operatórios de avaliação funcional. Orientavam o paciente sobre exercícios de fortalecimento a serem realizados antes da cirurgia, para que pudesse manter ou até mesmo ganhar massa muscular nos extensores do quadril e joelho, facilitando a reabilitação física pós-operatória.

O critério de escolha da abordagem cirúrgica foi feito de acordo com a experiência do cirurgião. Cada médico padronizou o uso de apenas uma via de acesso ao quadril a ser realizada em todos os seus pacientes e a randomização ocorreu devido ao critério aleatório de agendamento das consultas ambulatoriais pré cirúrgicas com os cirurgiões.

A anestesia preferencial foi a raquianestesia sem o uso de morfina complementada com infiltração peri capsular e peri acetabular realizada ao final do procedimento pelo cirurgião com 1ml por quilo da seguinte solução: 70ml de Soro Fisiológico Cloreto de Sódio 0,9%, 30 ml de Ropivacaína 7,5mg/ml, 1 ml de Adrenalina 1mg/ml e 1 ml de Clonidina 150mcg/ml. Caso o paciente apresenta-se contraindicação para raquianestesia a anestesia geral era realizada.

O uso da infusão de hemoderivados foi criteriosamente avaliado levando-se em consideração a percepção do cirurgião sobre a perda sanguínea durante o procedimento cirúrgico, o cálculo da perda de fluidos aspirados e as condições clínicas foram monitoradas pelo anestesiologista.

O encaminhamento pós-operatório para a UTI foi restrita apenas aos pacientes que apresentaram instabilidade hemodinâmica, necessidade de infusão de hemoderivados ou alterações na monitorização cardiológica durante a cirurgia.

A percepção da dor na recuperação pós-anestésica foi avaliada pela equipe anestésica. Analgésicos simples ou anti-inflamatórios foram prescritos e opioides foram deixados apenas como opção de resgate.

O paciente foi recebido da Recuperação Pós-Anestésica, avaliado pela equipe de enfermagem e a nutricionista prescrevia a dieta, evitando jejum prolongado.

A equipe de fisioterapia avaliou o paciente e o orientou sobre a necessidade de deambulação precoce para evitar comorbidades pós-operatórias como TVP.

A profilaxia de TVP era instaurada com Enoxaparina Sódica 40mg Subcutânea 1 vez ao dia durante o período de internação na enfermaria.

Os cirurgiões realizavam consultas médicas em conjunto com o fisioterapeuta para dar suporte e orientações iniciais de marcha no mesmo dia ou no dia seguinte a cirurgia. Os critérios para o início de marcha eram a capacidade do paciente sentar na cama, conseguir fazer transferência sozinho com o auxilio do andador para a cadeira, ficar em pé com o apoio do andador sem dor.

Caso tenha sido utilizado dreno pós-operatório, este foi retirado preferencialmente em até 24 horas.

As orientações de alta hospitalar foram fornecidas pela equipe multidisciplinar, enfatizando os seguintes aspectos: Incentivar o paciente, familiar e/ou cuidador a falar sobre suas preocupações com o cuidado domiciliar, continuar a reabilitação em casa com ênfase nas restrições pós-cirúrgicas como não dobrar o quadril mais de 90 graus, não cruzar a perna operada sobre a outra, não girar internamente o pé do lado operado.

Os pacientes eram liberados para alta hospitalar se atingissem os seguintes critérios: estar sem dor, deambulando sozinho com ou sem o uso de andador, ter capacidade de se sentar na cama sozinho, ter defecado e urinado espontaneamente, o curativo da incisão cirúrgica tinha que estar seco e limpo, parâmetros de pressão arterial, frequência cardíaca e temperatura normais.

Foram receitados analgésicos simples para casa como Dipirona 1g de 6/6h se dor e Paracetamol 750mg de 6/6h se dor e caso houvesse dor moderada ou intensa deixamos Tramadol 50mg de 6/6h se dor moderada ou forte. Foi prescrito também a profilaxia de TVP com Ácido Acetil Salicílico (AAS) 100mg via oral por 30 dias.

As avaliações quanto as complicações pós-operatórias abrangeram o pós-operatório imediato até 3 meses de acompanhamento ambulatorial.

Reuniões semanais entre os integrantes da equipe multidisciplinar foram realizadas com a intenção de uniformizar as informações e distribuir tarefas a cumprir para adequar os conceitos do PRR a realidade brasileira.

Durante as reuniões para que o PRR fosse implantado, as dúvidas eram discutidas em conjunto, estabelecendo uma meta de ações a serem cumpridas. O engajamento pessoal de cada profissional foi fundamental para que essas ações fossem implementadas pois a comunicação desses novos conceitos aos respectivos profissionais de cada especialidade envolvida, ficava a cargo do profissional participante do Grupo de Implementação do PRR ( Fig. 1 ).

**Fig. 1:** Cuidados pré, intra e pós-operatórios para implantação do PRR. (Adaptado de Soffin EM, YaDeau JT. Enhanced recovery after surgery for primary hip and knee arthroplasty: a review of the evidence. Br J Anaesth. 2016 Dec;117)

## Análise Estatística

Foram realizadas análises descritivas onde os dados quantitativos que apresentaram distribuição normal foram apresentados com médias acompanhadas dos respectivos desvios padrão. Os dados que não apresentaram distribuição normal foram apresentados com medianas e intervalos interquartis IQ (25%-75%). As variáveis categóricas foram apresentadas com freqüência e porcentagem. A distribuição normal em cada grupo e a homogeneidade das variâncias entre os grupos foram avaliadas, respectivamente, com o teste de Shapiro-Wilk e o teste de Levene.

Os testes de Wilcoxon para uma amostra e Kruskal Wallis foram utilizados para análise das variáveis numéricas para comparações múltiplas foi utilizado o teste de Dunn.

Para comparação de proporções, recorreu-se ao teste de Qui Quadrado ou exato de Fisher quando foi necessário.

Foi utilizado o valor de significância estatística menor ou igual a 5% (p ≤ 0,05) para todas as análises.

A modelagem estatística e os testes foram realizados com o software SPSS versão 21.0.

## Resultados

Todos os dados do estudo de 98 pacientes submetidos à artroplastia total do quadril no período de dezembro de 2018 a março de 2020 foram registrados em prontuários e apresentavam média de idade de 62,8 anos (34 a 81), sendo 56 homens e 43 mulheres divididos nos seguintes grupos etários demonstrados na Fig. 2 . 94 pacientes (95%) foram operados por osteoartrose primária e 4 por osteoartrose secundária a outras patologias, com as seguintes comorbidades associadas ( Fig. 3 ).

**Fig. 2:** Grupo etário dos pacientes da amostra.

**Fig. 3:** Incidência de comorbidades na população estudada. DIC – Doença infecto contagiosa, CV – Doença Cardiovascular, HAS- Hipertensão Arterial Sistêmica, DM2–Diabetes Mellitus tipo 2

Dos 98 pacientes, 54 foram operados pelo acesso AL, 35 pelo ADA e 9 pelo AP.

Na população estudada, tivemos 14 (14,29%) dos pacientes classificados como ASA1; 71 (72,45%) dos pacientes classificados como ASA 2 e 13 (13,27%) dos pacientes classificados como ASA 3 como mostra a Fig. 4 .

**Fig. 4:** Distribuição dos pacientes segundo a classificação ASA da American Society of Anestesiology.

A Taxa Média de Permanência Hospitalar foi de 2,8 dias ( Fig. 4 ) com 3 (3,06%) pacientes ficando 1 dia, 45 (45,92%) ficando 2 dias, 30 (30,61%) ficando 3 dias, 15 (15,41%) ficando 4 dias, 2 (2,04%) 5 dias, 2 (2,04%) 6 dias e 1 (1,02%) permanecendo 12 dias conforme a Fig. 5 .

**Fig. 5:** Permanência hospitalar: 3 pacientes ficaram 1 dia, 45 pacientes ficaram 2 dias, 30 pacientes ficaram3 dias, 15 pacientes ficando 4 dias, 2 pacientes ficaram 5 dias, 2 pacientes ficaram 6 dias e 1 paciente 12 dias

A Taxa Média de Permanência Hospitalar para ADA foi de 2,1 dias, 3 dias para AL e 4,11 dias para AP como visto na Fig. 6 .

**Fig. 6:** Taxa de permanência hospitalar de 2,1 dias para via anterior, 3,0 dias para via anterolateral e 4,11 dias para via posterior

O tempo médio de cirurgia foi de 90 minutos, 4 (4%) pacientes necessitaram de transfusão de hemoderivados, 19 (19%) dos pacientes foram encaminhados à UTI no pós-operatório sendo que 14 (76%) permaneceram 1 dia e 5 (26%) 2 dias.

Dos 35 pacientes operados pelo ADA, nenhum foi para a UTI no pós-operatório, enquanto 19 (30,15%) operados por outras vias foram para a UTI, conforme Fig. 7 .

**Fig. 7:** Nenhum paciente operado por via anterior foi para a UTI no pós-operatório. 30,15% dos pacientes operados pelas outras vias foram para a UTI e 69,85% não foram

Da amostra total de 98 pacientes, 4 (4,08%) necessitaram receber transfusão de hemoderivados durante o procedimento cirúrgico, 1 (1,02%) operado pelo ADA e 3 (3,06%) pelo AL. Não houve pacientes que receberam transfusões após o término do procedimento cirúrgico, conforme Fig. 8 abaixo.

**Fig. 8:** Do total da amostra de 98 pacientes, 4(4,08%) receberam transfusão de hemoderivados sendo 1(1,02%) operado por via anterior e 3(3,06%) por via lateral.

Durante o período de internação o estímulo a deambulação precoce foi seguido conforme a metodologia e todos os pacientes tiveram alta hospitalar deambulando com o auxílio do andador.

Quanto às complicações pós-operatórias imediatas durante o período de internação, não tivemos casos de trombose venosa profunda (TVP), embolia pulmonar (TEP) ou lesão neurológica, 19 (19,39%) pacientes tiveram sangramento pós-operatório com necessidade de troca de curativo, 4 (4,08%) necessidade de transfusão sanguínea, 2 (2,04%) pacientes apresentaram instabilidade do implante verificadas no período de internação, 1 por via anterolateral e 1 por via posterior e foram tratados de modo conservador, 1(1,02%) paciente teve fratura durante a cirurgia sendo realizada cerclagem do fêmur e 1(1,02%) paciente faleceu por complicações cardíacas, como pode ser observado na Fig. 9 .

**Fig. 9:** Total da amostra 98 paciente, 19(19,39%) dos pacientes apresentaram sangramento no pós-operatório imediato, 4(4,08%) necessitaram de transfusão de hemoderivados, 2(2,04%) apresentaram instabilidade do implante, 1(1,02%) tiveram fratura do fêmur, 1(1,02%) veio a óbito. Não houveram casos de Trombose Venosa Profunda (TVP), Tromboembolismo Pulmonar (TEP) ou Lesão de Nervo Periférico.

No acompanhamento pós-operatório em até 3 meses tivemos as seguintes complicações: 2(2,04%) pacientes apresentaram infecção superficial na via de acesso tratados com antibioticoterapia oral, 1(1,02%) paciente teve uma fratura de fêmur sem desvio por queda da própria altura, 2 meses após o procedimento cirúrgico inicial e foi tratado de modo conservador e 1(1,02%) paciente teve TVP diagnosticada no membro operado 15 dias após o procedimento cirúrgico, no entanto foi constatado que a paciente não tomou a profilaxia recomendada conforme pode ser observado na Fig. 10 .

**Fig. 10:** Complicações pós-operatórias em até 3 meses. Tivemos 2 (2,04%) de infecção superficial da ferida operatória, 1 (1,02%) de fratura de fêmur e 1 (1,02%) caso de TVP

## Discussão

A nossa Taxa de Permanência Hospitalar (TPH) geral foi de 2.8 dias, sendo 2.1 dias para via anterior, 3.0 dias para via anterolateral e 4.1 dias para via posterior.

Segundo Marcio de Castro Ferreira, em seu artigo “Artroplastia total de joelho e quadril: A preocupante realidade assistencial do Sistema Único de Saúde brasileiro”, a taxa de permanência hospitalar média nacional para artroplastia total do quadril eletiva é de 7,1 dias 2 ( Fig. 11 ).

**Fig. 11:** A taxa de permanência de todos os pacientes da amostra foi de 2,8 dias e da média nacional é de 7,1 dias (p < 0,001).

No geral, há pouca evidência para apoiar o uso de educação pré-operatória estruturada para reduzir eventos adversos pós-operatórios, melhorar a dor, facilitar a recuperação funcional ou reduzir o tempo de internação. No entanto, uma redução significativa na ansiedade pré-operatória pode ser alcançada. A educação pré-operatória pode beneficiar mais pacientes com depressão, ansiedade, expectativas irreais ou com suporte social limitado. 7 8 9

A redução do tempo de internação está consistentemente associada ao uso de anestesia neuroaxial (bloqueio anestésico) em comparação com o uso de anestesia geral. Em um estudo retrospectivo multicêntrico associando o uso de anestesia geral com um aumento de 8,5 vezes no risco de dor pós-operatória moderada a intensa e um aumento de 2,5 vezes no risco de dor pós-operatória persistente, para artroplastia de quadril e joelho. 10 11 Esses dados fornecem motivação adicional para o uso de anestesia neuroaxial ou bloqueio de plexo.

A raquianestesia sem o uso de opioide complementada com analgesia por infiltração local (ALI) é uma técnica recente para analgesia pós-operatória precoce após artroplastia de joelho e quadril, e foi administrada pelos cirurgiões logo após o término do procedimento cirúrgico, proporcionando 6–12 horas de alívio da dor de modo que os pacientes se beneficiam da analgesia multimodal e de opioides orais apenas se necessário. 12 13

Nossos resultados com uma amostra total de 98 pacientes demonstraram que 19 (19,39%) apresentaram sangramento no pós-operatório imediato com a necessidade da troca do curativo cirúrgico, 4 (4,08%) necessitaram de transfusão de hemoderivados, 2 (2,04%) tiveram instabilidade do implante com sinais de subluxação da prótese, um paciente operado por via anterolateral e um paciente operado por via posterior sendo ambos tratados de modo conservador e acompanhamento ambulatorial, 1 (1,02%) teve fratura de fêmur durante o procedimento cirúrgico sendo realizada a fixação com cerclagem com fios de aço, 1 (1,02%) faleceu por complicações cardíacas.

Os resultados obtidos corroboram Starks et al. que aplicou o PRR em pacientes submetidos a artroplastia total de quadril ou joelho, observou que a taxa de mortalidade para artroplastia total de joelho diminuiu de 0,44 para 0,07%. O trabalho de Starks pode ser visto como um modelo inicial para PRR em cirurgia ortopédica. 5

Diretrizes nutricionais agora permitem a ingestão de líquidos até 3h antes da indução da anestesia e jejum de 6h para alimentos sólidos. Além do tempo de jejum reduzido, o PRR recomenda que os pacientes consumam até 300 ml de uma bebida clara rica em carboidratos 3 horas antes da cirurgia, com o objetivo de apresentar o paciente para a cirurgia em estado metabolicamente “alimentado”, evitando o catabolismo. 14 15 16 17

Na nossa amostra não tivemos nenhum evento de TVP ou TEP durante o período de internação. A mobilização precoce é um componente chave do PRR. Os efeitos fisiológicos adversos do repouso prolongado no leito incluem aumento da resistência à insulina, miopatia, redução da função pulmonar, comprometimento da oxigenação tecidual e aumento do risco de tromboembolismo pulmonar. 18 Pua et al. 19 demonstraram uma redução significativa do tempo de internação em 1,8 dia, quando os pacientes caminhavam em até 24 horas após a cirurgia. A mobilização precoce após artroplastia do joelho também está associada à melhora da recuperação funcional e menor incidência de Trombose Venosa Profunda (TVP). 20 21

O estímulo a deambulação precoce foi seguido conforme os critérios da metodologia e todos os pacientes receberam alta hospitalar deambulando com o auxílio do andador e recomendações de segurança realizada pela equipe de fisioterapia e enfermagem.

Na nossa amostra, somente 4 (4,08%) pacientes receberam transfusão de hemoderivados durante o procedimento cirúrgico. Uma estratégia que preserve a necessidade de administração de sangue é crucial para o sucesso do PRR. A transfusão de sangue alogênico está associada à imunomodulação e sobrecarga de volume sistêmico. 22

Embora inicialmente adotado em cirurgias ortopédicas para artroplastias primárias de quadril e joelho, o PRR vem sendo cada vez mais aplicado a outros procedimentos ortopédicos, trazendo benefícios aos pacientes e reduzindo os custos dos procedimentos. 23 24 25 26 27

Recentemente um artigo da Mayo Clinic, importante hospital referência em cirurgias ortopédicas nos Estados Unidos, relatando os benefícios obtidos em cirurgias de artroplastia conciliadas com o PRR, onde os pacientes submetidos à cirurgia tiveram menos efeitos colaterais relacionados aos opióides, menos complicações pós-operatórias, menor tempo de internação e maior economia de custos para a clínica. 28 29

Acreditamos que os motivos para a alta precoce destes pacientes clinicamente estáveis incluem vários aspectos, entre eles a orientação pré operatória que diminui a ansiedade quanto ao procedimento e estabelece que não é necessário ficar internado por um longo período para se recuperar adequadamente, o uso da técnica mista de anestesia entre a raquianestesia e a anestesia por infiltração local proporcionando mais conforto ao paciente por um tempo maior contribuindo para o mesmo sair da fase inflamatória aguda do pós-operatório, o uso adequado de hemoderivados contribuindo para se evitar um rebaixamento fisiológico do paciente devido a transfusão de elementos exógenos, o incentivo a mobilização precoce contribuindo para o retorno das funções gastrointestinais, circulação periférica dos membros e circulação profunda da região operada aumentando a perfusão tecidual com oxigênio e contribuindo para a diminuição do processo inflamatório e por fim a ausência de complicações como TEP e TVP no pós-operatório imediato.

## Conclusão

A implantação do PRR exige que uma equipe multidisciplinar composta por cirurgiões, anestesiologistas, enfermeiros, fisioterapeutas, nutricionistas e assistentes sociais trabalhem em conjunto para que as etapas sejam cumpridas e as necessidades sejam implementadas, verificadas e avaliadas constantemente para atingir um índice aceitável, cumprindo todas as ações planejadas.

O PRR só funciona bem quando todas as partes contribuem para o resultado global e reuniões semanais de acompanhamento e planejamento, não só na fase de implementação, mas também na execução diária, são essenciais para que o projeto seja executado de forma correta e eficaz.

O PRR tem a capacidade de reduzir o tempo de internação hospitalar, diminuir a necessidade de uso de UTI, uso de hemoderivados e analgésicos de alto custo, contribuindo para uma recuperação precoce e mais eficiente do paciente, bem como a redução de custos hospitalares e sociais. custos no retorno desse paciente às suas atividades de vida diária.

Chamo a atenção para a efetividade do PRR em um Hospital Público e enfatizar a importância do trabalho multidisciplinar e de auditoria de dados para melhor fundamentar as conclusões.
